# The effects of glomerular and tubular renal progenitors and derived extracellular vesicles on recovery from acute kidney injury

**DOI:** 10.1186/s13287-017-0478-5

**Published:** 2017-02-07

**Authors:** Andrea Ranghino, Stefania Bruno, Benedetta Bussolati, Aldo Moggio, Veronica Dimuccio, Marta Tapparo, Luigi Biancone, Paolo Gontero, Bruno Frea, Giovanni Camussi

**Affiliations:** 1 Department of Medical Sciences and Molecular Biotechnology Center, University of Torino, Corso Dogliotti 14, Torino, 10126 Italy; 2 Department of Molecular Biotechnology and Health Sciences and Molecular Biotechnology Center, University of Torino, Torino, Italy; 3 Department of Surgical Sciences, Città della Salute e della Scienza, University of Turin, Torino, Italy

**Keywords:** Glomerular mesenchymal stromal cells, Extracellular vesicles, Ischemia-reperfusion injury, Renal regeneration

## Abstract

**Background:**

Mesenchymal stromal cells (MSCs) and renal stem/progenitors improve the recovery of acute kidney injury (AKI) mainly through the release of paracrine mediators including the extracellular vesicles (EVs). Several studies have reported the existence of a resident population of MSCs within the glomeruli (Gl-MSCs). However, their contribution towards kidney repair still remains to be elucidated. The aim of the present study was to evaluate whether Gl-MSCs and Gl-MSC-EVs promote the recovery of AKI induced by ischemia-reperfusion injury (IRI) in SCID mice. Moreover, the effects of Gl-MSCs and Gl-MSC-EVs were compared with those of CD133^+^ progenitor cells isolated from human tubules of the renal cortical tissue (T-CD133^+^ cells) and their EVs (T-CD133^+^-EVs).

**Methods:**

IRI was performed in mice by clamping the left renal pedicle for 35 minutes together with a right nephrectomy. Immediately after reperfusion, the animals were divided in different groups to be treated with: Gl-MSCs, T-CD133^+^ cells, Gl-MSC-EVs, T-CD133^+^-EVs or vehicle. To assess the role of vesicular RNA, EVs were either isolated by floating to avoid contamination of non-vesicles-associated RNA or treated with a high dose of RNase. Mice were sacrificed 48 hours after surgery.

**Results:**

Gl-MSCs, and Gl-MSC-EVs both ameliorate kidney function and reduce the ischemic damage post IRI by activating tubular epithelial cell proliferation. Furthermore, T-CD133^+^ cells, but not their EVs, also significantly contributed to the renal recovery after IRI compared to the controls. Floating EVs were effective while RNase-inactivated EVs were ineffective. Analysis of the EV miRnome revealed that Gl-MSC-EVs selectively expressed a group of miRNAs, compared to EVs derived from fibroblasts, which were biologically ineffective in IRI.

**Conclusions:**

In this study, we demonstrate that Gl-MSCs may contribute in the recovery of mice with AKI induced by IRI primarily through the release of EVs.

**Electronic supplementary material:**

The online version of this article (doi:10.1186/s13287-017-0478-5) contains supplementary material, which is available to authorized users.

## Background

Acute kidney injury (AKI) is a major clinical disorder that affects more than 20% of hospitalized patients. Furthermore, the adjusted odds ratio for hospital mortality in patients that develop AKI stands at 4:1 [1, 2]. Out of all the causes of AKI, ischemia-reperfusion injury (IRI) signifies to be one of the most important, as it is characterized by severe tubular damage associated with rapid worsening of the renal function [3]. Moreover, IRI may subsequently develop into chronic kidney disease (CKD), mainly due to fibroblast proliferation and the deposition of extracellular matrix.

Regeneration of injured tubular and endothelial cells after AKI may occur through multiple mechanisms including the de-differentiation of surviving resident cells [4–8] and/or the intrinsic ability of resident progenitor cells to proliferate and differentiate into new renal cells [9–14]. A population of resident renal progenitor cells expressing the human stem cell antigen CD133 and the embryonic renal marker *PAX2* has been identified in the tubular compartment [9]. Furthermore, Sagrinati et al. reported the presence of renal progenitor cells characterized by the co-expression of CD133 and CD24 within the Bowman’s capsule [11]. Subsequently, CD133^+^ progenitor cells were also found to be present in different compartments of the nephron [9, 11–13, 15]. Several authors demonstrated that these progenitor cells could contribute towards kidney repair after injury in different murine models of AKI [9, 10, 12, 16]. In addition, over the last decade, numerous studies performed in animal models of AKI and CKD have reported the beneficial effects of mesenchymal stromal cells (MSCs) not only in the recovery of renal function after IRI, but also in reducing the progression of the chronic damage that followed [17–23]. The mechanism by which MSCs exert these effects seems to be primarily due to a paracrine action on the target cells rather than transdifferentiation into resident cells [24–27]. It is well known that MSCs release soluble factors which promote the recovery of damaged renal cells [28–31]. Among these factors, extracellular vesicles (EVs) have been implicated to play a role in the paracrine actions of MSCs [32]. EVs are circular cellular membrane fragments that are released from a given cell type and influence target cells by delivering proteins, lipids and nucleic acids [33–37]. Amidst various types of nucleic acids transported by EVs, the capacity of mRNAs to induce epigenetic changes in target cells in murine models of AKI using MSC-derived EVs has been well demonstrated by several authors [38–40]. In addition, several studies have also demonstrated the presence of microRNAs (miRNA) in EVs that could be transferred to the target cells modulating their phenotype [36, 41]. Other than nucleic acids, proteins carried by EVs also have significant effects on target cells. For instance, Sallustio et al. recently reported that the protein decorin carried by EVs from adult renal stem/progenitor cells improved the survival of tubular epithelial cells in an in vitro toxic AKI model [42].

MSCs are stem cells that have been reported to reside in almost all organs. Furthermore, they have also been identified to be present within the glomeruli of both mice and human [43, 44]. However, their role in the repair of kidney injury is still unknown.

The aim of the present study was to evaluate whether the MSCs derived from human glomeruli (Gl-MSCs) and their EVs (Gl-MSC-EVs) promote the recovery of AKI induced by IRI in SCID mice. Furthermore, the effects of Gl-MSCs and Gl-MSC-EVs were compared with those of CD133^+^ progenitor cells isolated from human tubules of the renal cortical tissue (T-CD133^+^ cells) and their EVs (T-CD133^+^-EVs).

## Methods

### Isolation and characterization of different resident renal stem/progenitor cell populations

Normal portions of renal cortex were obtained from surgically removed kidneys of cancer patients with informed consent, obtained in accordance with the Declaration of Helsinki and after approval by the ethic committee of the Azienda Ospedaliera Universitaria, Città della Salute e della Scienza, Torino (N. 168/2014). After dissection and passage through a graded series of mesh (60 and 120 mesh per inch), T-CD133^+^ cells were isolated form the tubular fraction by magnetic cell sorting, using the MACS system (Miltenyi Biotec, Auburn, AL, USA). T-CD133^+^ cells were cultured and expanded as previously described [9]. The glomeruli were recovered from the top of the 120-mesh sieve and collected at the bottom of a conical tube by low-speed centrifugation (300 g, 5 minutes). To obtain Gl-MSCs, the visceral layer of the Bowman’s capsule was removed mechanically by several rounds of aspirations/expulsions using a 10-ml pipette, followed by an enzyme digestion for 2 minutes with collagenase I (Sigma-Aldrich, St. Louis, MO. USA). The decapsulated glomeruli were then collected by low-speed centrifugation and transferred to a fibronectin-coated T25 flask and cultured as previously described [44].

Cytofluorimetric analyses were performed as previously described [9, 44] using the following antibodies: anti-CD105, -CD29, -CD73, -CD44, -CD133, -CD146, -CD24, -CD31, -CD90, -CD45 (all from Mitenyi Biotech, Auburn, AL, USA). T-CD133^+^ cells co-expressed CD73, CD44, CD29, CD90, CD146 and CD24; no expression of CD45, CD31 and CD105 was detected (Table 1). Gl-MSCs were positive for surface markers characteristic of MSCs, such as CD29, CD73, CD105, CD146, CD44, CD90 and for CD24. Gl-MSCs were negative for CD133 and for specific hematopoietic (CD45) and endothelial markers (CD31) (Table 1). Human dermal fibroblasts were used as control (Lonza, Basel, Switzerland). FACS analyses indicated that the fibroblasts expressed CD73, CD44, CD29 and CD24 (Table 1).

**Table 1:** Phenotype of different renal stem/progenitor cell populations

	T-CD133+	Gl-MSCs	Fibroblasts
Positive	CD90 CD73 CD44 CD29 CD24 CD133 CD146	CD90 CD73 CD44 CD29 CD24 CD105 CD146	CD73 CD44 CD29 CD24
Negative	CD45 CD31 CD105	CD45 CD31 CD133	CD45 CD133 CD90 CD31 CD146 CD105

*T-CD133* ^*+*^ CD133^+^ progenitor cells isolated from human tubules of the renal cortical tissue, *Gl-MSCs* Resident population of MSCs within glomeruli

### Isolation and characterization of EVs derived from Gl-MSCs and T-CD133^+^ cells

Briefly, healthy stem/progenitor cells were incubated in serum-free RPMI 1640 overnight at 37 °C. Post incubation, the cell supernatant was collected and centrifuged at 3,000 g for 20 minutes to remove cell debris and apoptotic bodies. This was followed by ultracentrifugation at 100,000 g for 2 hours at 4 °C (Beckman Coulter Optima L-90 K, Fullerton, CA, USA) to pellet the EVs [38, 45]. The EVs obtained were then resuspended in RPMI containing 1% dimethyl sulfoxide (DMSO, Sigma-Aldrich) and stored at -80 °C until further use.

In order to trace EVs by fluorescent microscopy post in vivo injection, they were labelled with PKH26 dye (a red fluorescent aliphatic cromophore intercalating into lipid bilayers) as per manufacturer’s instructions (PKH26 dye, Sigma-Aldrich). After labelling, the EVs were washed with PBS by ultracentrifugation at 100,000 g for 2 hours at 4 °C.

To separate membrane-enclosed vesicles from aggregates of protein and other molecules (e.g. extra-vesicle RNAs), EVs were isolated by a floating process into a gradient, as described previously [46, 47]. Briefly, EVs from 80 million Gl-MSCs were resuspended in 1.35 ml of buffer (0.25 M sucrose, 10 mM Tris pH 8 and 1 mM EDTA), transferred to a SW55Ti rotor tube (Beckman Coulter) and mixed with 60% stock solution of Optiprep (Sigma-Aldrich) in a 1:1 ratio. Next, 1.2 ml of 20% Optiprep solution was layered on top, followed by 1.1 ml of 10% Optiprep solution. The tubes were then ultracentrifuged at 350,000 g for 1 hour at 4 °C. Five fractions of 1 ml were collected from the top of the tubes, each fraction was diluted in 20 ml PBS and ultracentrifuged at 100,000 g for 1 hour at 4 °C to pellet the EVs. Nanosight analyses (not shown) indicated that fraction 2 contained vesicles with a similar size range of exosomes, and therefore that fraction was used for the in vivo experiments. Treatment with 0.2 μg/mL RNase did not inactivate RNA present in the floating fraction (0.9 ng/mL untreated; 1.0 ng/mL RNase floating EVs).

To characterize the phenotype of EVs, cytofluorimetric analysis was performed as previously described [45]. Briefly, EVs were incubated for 15 minutes at 4 °C with the following antibodies: anti-CD24, -CD29, -CD146, -CD133 (Miltenyi Biotech), -CD107, (Becton Dickinson, Franklin Lakes, NJ, USA) and SSEA4 (R&D Systems, Minneapolis, MN, USA). Mouse nonimmune isotypic IgG (Miltenyi Biotech) was used as control. For each preparation of EVs, 5000 particles were acquired using the Guava easyCyte™ Flow Cytometer (EMD Millipore, Billerica, MA, USA) and analysed with the InCyte™ software.

EV size and concentration were measured by the NanoSight LM10 instrument (NanoSight Ltd, Amesbury, UK) equipped with a 405 nm laser and the nanoparticle tracking analyses (NTA) software version 2.3. Three videos of 30 seconds duration were recorded in order to perform the analyses.

### RNase treatment, RNA isolation and analyses

For selected experiments, EVs were treated with 5 μg/ml of RNase for 1 hour at 37 °C. The reaction was stopped by adding 1 U of RNase inhibitor (Ambion, Austin, TX, USA) per 5 ng of RNAse used, and the EVs washed and pelleted by ultracentrifugation (2 hours at 100,000 g). RNA was extracted from cells and EVs using the mirVana RNA isolation kit (Applied Biosystems, Foster City, CA, USA) as per the manufacturer’s protocol. The quantification of RNA was performed using the Nanodrop spectrophotometer (ND-1000; Nanodrop, Wilmington, DE, USA), and the quality of RNA was assessed by capillary electrophoresis on an Agilent 2100 Bioanalyzer (Agilent Technologies, Santa Clara, CA, USA) using the Total Eukaryotic Pico RNA kit.

### miRNA expression and target gene enrichment analysis

To investigate miRNAs carried by Gl-MSC-EVs and fibroblast EVs, 60 ng of input RNA was run on TaqMan™ Array Human MicroRNA A card (Thermo Fisher Scientific, Waltham, MA, USA), that profiles 365 human mature miRNAs by qRT-PCR. Raw Ct values were analysed using the SDS 2.3 software with automatic baseline and threshold settings. The data were expressed as Relative Quantification (RQ) using the ΔΔCt method and RNU6B was used as housekeeping gene for normalization. To identify miRNA specifically expressed and upregulated in Gl-MSC-EVs, we applied different Ct cutoff levels for fibroblast EVs (Ct >35, not expressed) and Gl-MSC-EVs (Ct <35, expressed). Target prediction and biological process enrichment analysis was conducted using Funrich V3 analysis tool [48]. Only the biological process of target genes with a *p* value <0.05 were considered as significantly enriched.

### Animal model of monolateral kidney IRI

All procedures were approved by the Ethics Committee of the University of Torino and conducted in accordance with the National Institute of Health Guide for the Care and Use of Laboratory Animals. Male SCID mice (Charles River Laboratories, Wilmington, MA, USA) aged 7 to 8 weeks and weighing 22 to 26 g were anesthetized with an intramuscular (i.m.) injection of zolazepam 80 mg/kg and xilazina 16 mg/kg. Postoperatively, the animals were closely monitored, and ketorolac (5 mg/kg) was administered as an analgesic if required. Under sterile conditions, a small mid laparotomy was made, the left kidney exposed and the renal pedicle was clamped for 35 minutes using a nontraumatic vascular clamp (Fine Science Tools, Foster City, CA, USA). Immediately after clamping the left renal pedicle, a right nephrectomy was performed using a subcapsular technique. Briefly, after isolation and ligation of the right kidney pedicle, the renal capsule was dissected and the renal parenchyma exposed. The nephrectomy was performed by the incision of the renal parenchyma leaving the capsule in situ. This procedure allows an accurate hemostasis to exclude bleeding due to the failure of the ligation of the renal pedicle. The laparotomy incision was temporarily closed during ischemia and the body temperature was maintained at 37 °C during the surgical intervention by placing the animals on a heating plate. Reperfusion of the kidney was then confirmed visually after removing the clamp. The abdominal incision was closed with a 6-0 silk suture. After surgical intervention, the mortality rate was approximately 10%.

In order to evaluate the effects of cells or EVs in AKI-IRI mice, the animals were divided in ten groups based on the different treatments (Table 2). The dose of EVs used was selected as the number of EVs produced overnight by 1 × 10^5^ cells under serum starvation (T-CD133^+^ -EVs: 480 × 10^6^/mouse; Gl-MSC-EVs: 400 × 10^6^/mouse; Gl-MSC-EV-float: 400 × 10^6^/mouse; F-EVs: 230 × 10^6^/mouse). For all the experiments, cells cultured up to passage 6 were detached by trypsin (Sigma-Aldrich), washed and resuspended in PBS (Lonza). The cells were injected intravenously (120 μl injecting volume, i.v*.*) through the tail vein. All the animals were sacrificed at day 2 after surgery. For bio-distribution analysis, mice were sacrificed at 1, 6 and 24 hours after surgery.

**Table 2:** Schematic representation of the groups of mice used in the study

	Treatment	Doses	No. animals	Time of sacrifice
	Normal	-	5	48 h
		-	5	3 weeks
	Sham-operated	-	5	48 h
	IRI-CTL (vehicle)	-	8	48 h
		-	6	3 weeks
Cells	IRI-Gl-MSC	10^5^ cells	8	48 h
		10^5^ cells	6	3 weeks
	IRI-T-CD133^+^	10^5^ cells	8	48 h
		10^5^ cells	6	3 weeks
EVs	IRI-Gl-MSC-EVs	400 × 10^6^ EVs^*^	8	48 h
	IRI-Gl-MSC-EVs-float	400 × 10^6^ EVs^*^	6	48 h
	IRI-RNase-Gl-MSC-EVs	400 × 10^6^ EVs^*^	8	48 h
	IRI-T-CD133^+^-EVs	480 × 10^6^ EVs^*^	8	48 h
	IRI-F-EVs	230 × 10^6^ EVs^*^	5	48 h

*IRI* ischemia-reperfusion injury, *CTL* control, *Gl-MSC* glomerular mesenchymal stromal cells, *T-CD133* ^*+*^ *-EVs* T-CD133^+^-derived EVs, *EVs* extracellular vesicles, *Gl-MSC-EVs* Gl-MSC-derived EVs, *Gl-MSC-EVs-float* Gl-MSC-derived EVs isolated through floating test, *RNase-Gl-MSC-EVs* Gl-MSC-derived EVs treated with 5 μg/ml RNase, *T-CD133* ^*+*^ CD133^+^ renal tubular cells, *F-EVs* fibroblast-derived EVs. *The doses of EVs used were selected as the number of EVs produced overnight by 10^5^ cells in serum starvation

In order to evaluate the long-term effect of Gl-MSCs and T-CD133^+^ cells on kidney function post IRI, and also to check the potential development of malignancy, supplementary experiments were performed in mice. The animals were divided into four groups according to the treatment administered intravenously immediately after surgery: normal mice (n = 5); IRI-CTL mice (n = 6) injected with vehicle alone; IRI-Gl-MSC mice (n = 6) injected with 1 × 10^5^ Gl-MSCs; IRI-T-CD133^+^ mice (n = 6) injected with 1 × 10^5^ T-CD133+ cells. The animals were sacrificed 3 weeks after intervention.

### Renal function

Blood samples for the measurement of plasma creatinine and blood urea nitrogen (BUN) were collected at 2 days after IRI from the different AKI groups and at 3 weeks from the long-term four groups of mice. Creatinine concentrations were determined using a colorimetric microplate assay based on the Jaffe reaction (Quantichrome Creatinine Assay, BioAssay Systems, Hayward, CA, USA). Creatinine levels that exceeded 0.3 mg/dl were considered abnormal (normal range in our laboratory: 0.1 to 0.3 mg/dl). BUN was measured by direct quantification of serum urea with a colorimetric assay kit according to the manufacturer’s protocol (Arbor Assays, Ann Arbor, MI, USA).

### Morphological studies

For renal histology, 5-μm-thick paraffin-embedded kidney sections were routinely stained with hematoxylin and eosin (Merck, Darmstadt, Germany). To evaluate the score of the AKI, luminal hyaline casts and the cell lose (denudation of tubular basement membrane) were assessed in non-overlapping fields (up to 28 for each section) using a x40 objective (high power field, HPF). The number of casts and tubular profiles showing necrosis were recorded in a single-blind fashion [49].

Immunohistochemistry to detect the proliferation of tubular cells was performed by BrdU incorporation as previously described [38]. Kidney sections were subjected to antigen retrieval and stained with anti-proliferating cell nuclear antigen (PCNA) (1:400, monoclonal anti-PCNA antibody; Santa Cruz Biotechnology, Dallas, TX, USA) or with anti-BrdU (1:200, Dako North America Inc., Carpinteria, CA, USA). Immunoperoxidase staining was performed using 1:300 dilution of anti-mouse horseradish peroxidase (HRP, Pierce, Rockford, IL, USA). Scoring for BrdU- and PCNA-positive cells was carried out by counting the number of positive nuclei per HPF (×40) in ten randomly chosen sections of the kidney cortex.

Confocal microscopy analysis (Zeiss LSM 5 Pascal; Carl Zeiss International, Oberkochen, Germany) was performed on frozen sections to assess the localization of PKH26-labelled EVs in different organs as described previously [38]. Nuclei were stained with Hoechst 33258 dye (Sigma-Aldrich).

### Statistical analysis

Statistical analysis was performed by using the *t* tests, analysis of variance (ANOVA) with Newmann–Keuls’ or ANOVA with Dunnett’s multiple comparison tests as appropriate. A *p* value of <0.05 was considered significant.

## Results

### Gl-MSCs cells are more effective in promoting the recovery of AKI compared to T-CD133+ cells

**Fig. 1:**
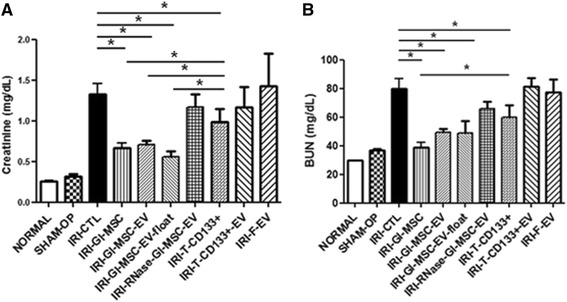
Effect of different renal stem/progenitor cells and their derived EVs in IRI mice: functional evaluation. **a** Serum creatinine and **b** BUN values at day 2 after IRI, in mice injected with vehicle alone (*IRI-CTL*), 1 × 10^5^ Gl-MSCs (*IRI-Gl-MSC*), 400 × 10^6^ EVs produced by Gl-MSCs (*IRI-Gl-MSC-EV*), 400 × 10^6^ EVs produced by Gl-MSCs and obtained by floating process (*IRI-Gl-MSC-EV-float*), 400 × 10^6^ EVs produced by Gl-MSCs and treated with RNase (IRI-RNase-Gl-MSC-EV), 1 × 10^5^ T-CD133+ cells (*IRI-T-CD133* ^*+*^), 480 × 10^6^ EVs produced by T-CD133^+^ cells (*IRI-T-CD133* ^*+*^ *-EV*), 230 × 10^6^ EVs derived from fibroblasts (*IRI-F-EV*), in healthy (normal) and in sham-operated SCID mice. Data are expressed as mean ± SD, ANOVA with Dunnett’s multiple comparison test were performed (^*^ *p* < 0.05)

**Fig. 2:**
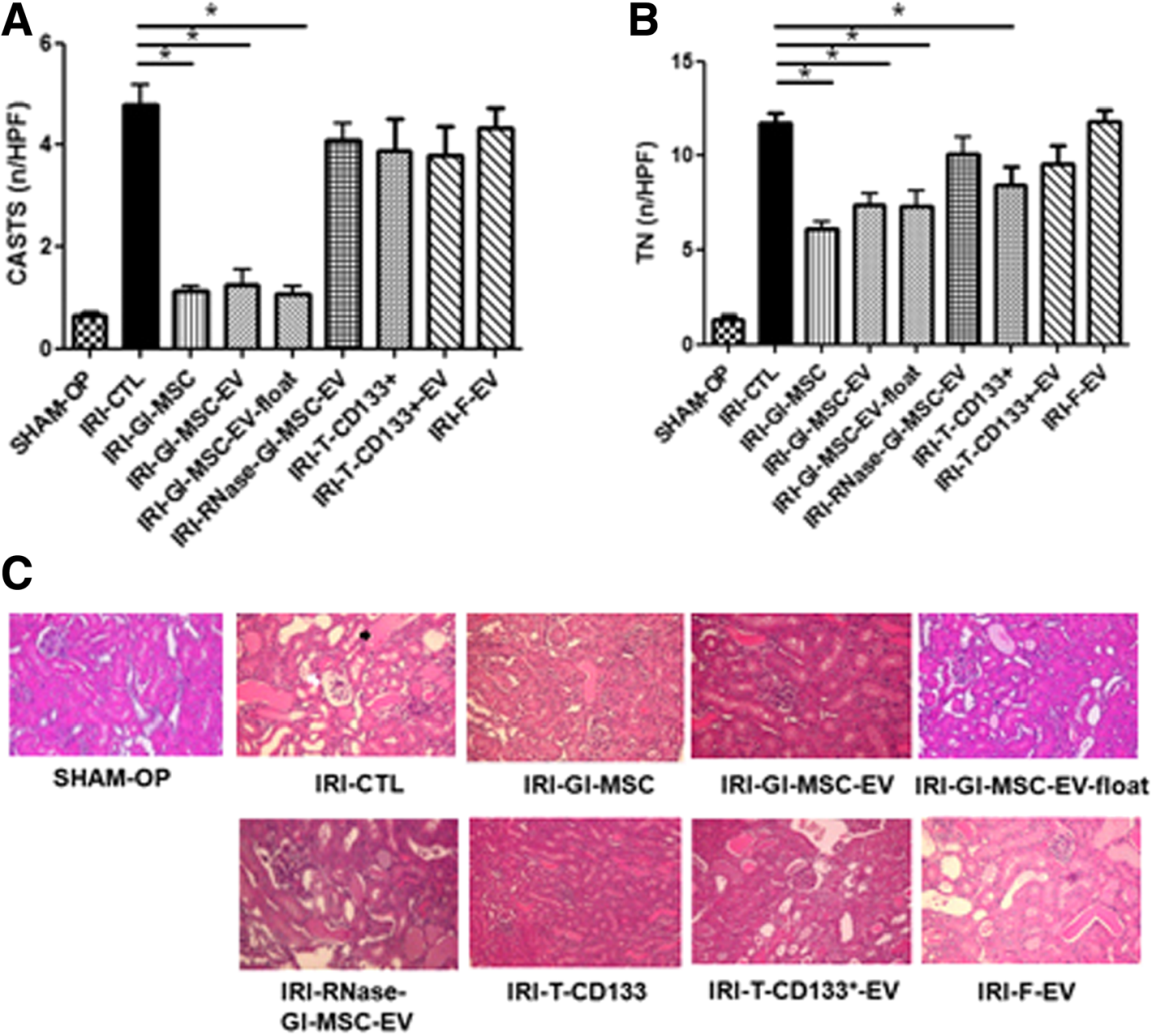
Effect of different renal stem/progenitor cells and their derived EVs in IRI mice: renal morphology. **a** The number of hyaline casts and **b** tubular necrosis (*TN*) observed under high power field (HPF: ×40) is expressed as mean ± SD. An ANOVA with Dunnett’s multiple comparison test was performed, (^*^ *p* < 0.05). **c** Representative micrographs of renal histology of: IRI mice injected with different treatments and sham-operated SCID mice. Original magnification: ×20. In the representative image related to IRI-CTL intratubular hyaline cast (*black arrow*) and a tubule with signs of necrosis (epithelial cell denudation and presence of intratubular cell debris) (*white arrow*) can be observed. *IRI-CTL* mice injected with vehicle alone, *IRI-Gl-MSC* mice injected with GI-MSC, *IRI-Gl-MSC-EV* EVs produced by Gl-MSCs, *IRI-Gl-MSC-EV-float* EVs produced by Gl-MSCs and obtained by floating process, *IRI-RNase-Gl-MSC-EV* EVs produced by Gl-MSCs and treated with RNase, *IRI-T-CD133* ^*+*^ T-CD133^+^ cells, *IRI-T-CD133* ^*+*^ *-EV* EVs produced by T-CD133^+^, *IRI-F-EV* EVs derived from fibroblasts

**Fig. 3:**
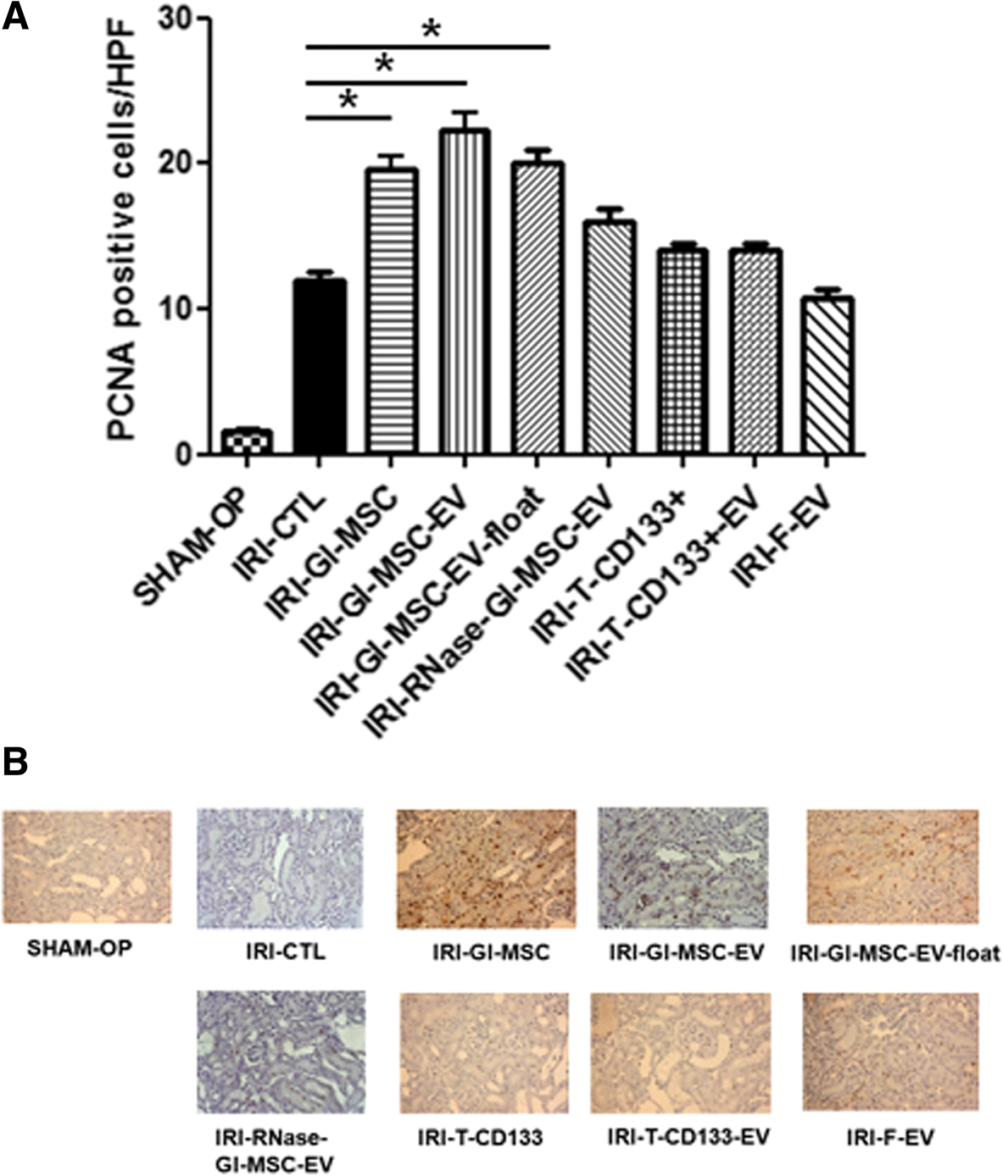
Renal cell proliferation in IRI-mice treated with different stem/progenitor cells and their derived EVs. **a** Quantification of PCNA-positive cells/high power field (HPF: ×40) was performed in renal sections of IRI mice injected with vehicle alone (*IRI-CTL*), 1 × 10^5^ Gl-MSCs (*IRI-Gl-MSC*), 400 × 10^6^ EVs from Gl-MSCs (*IRI-Gl-MSC-EV*), 400 × 10^6^ EVs from Gl-MSCs purified by floating process (*IRI-Gl-MSC-EV-float*), 400 × 10^6^ EVs from Gl-MSCs and treated with RNase (*IRI-RNase-Gl-MSC-EV*), 1 × 10^5^ T-CD133^+^ cells (*IRI-T-CD133* ^*+*^), 480 × 10^6^ EVs produced by T-CD133^+^ cells (*IRI-T-CD133* ^*+*^ *-EV*), 230 × 10^6^ EVs derived from fibroblasts (*IRI-F-EV*), and in sham-operated SCID mice. ANOVA with Dunnett’s multiple comparison test was performed, (^*^ *p* < 0.05). **b** Representative micrographs of PCNA staining preformed on section of kidneys of IRI-AKI mice injected with different treatments. Original magnification: ×40

Forty-eight hours after the induction of IRI, serum creatinine and BUN markedly increased in IRI mice compared to healthy and sham-operated mice (Fig. 1). Furthermore, histological analysis of the kidney revealed severe tubular damage characterized by tubular necrosis and presence of proteinaceous casts inside the lumen of tubules in IRI mice (Fig. 2). However, injecting mice with Gl-MSCs significantly reduced both functional and histological alterations observed in IRI mice, evaluated 48 hours after surgery (Figs. 1 and 2). Although treating IRI mice with T-CD133^+^ cells was associated with a significant improvement in renal function and morphology compared to control animals, the treatment was not as efficient in ameliorating the recovery after IRI as the treatment with Gl-MSCs cells. In addition, 48 hours after IRI induction, PCNA staining showed a significant increase in tubular cell proliferation in mice treated with Gl-MSCs compared to both control mice and mice treated with T-CD133^+^ cells (Fig. 3). No significant differences, however, were observed in the rate of apoptosis between animals treated with Gl-MSCs or T-CD133^+^ cells (data not shown).

In order to evaluate the long-term effects of Gl-MSCs and T-CD133^+^ cells, mice were sacrificed 3 weeks after intervention. Analysis of the results revealed no significant differences in the functional and histological markers of tubular damage between the control group and the animals treated with cells (data not shown). In addition, no signs of neoplastic lesions or mal-differentiation of the engrafted cells were detected in the renal parenchyma (data not shown).

### Characterization of EVs derived from Gl-MSCs and T-CD133^+^ cells

**Fig. 4:**
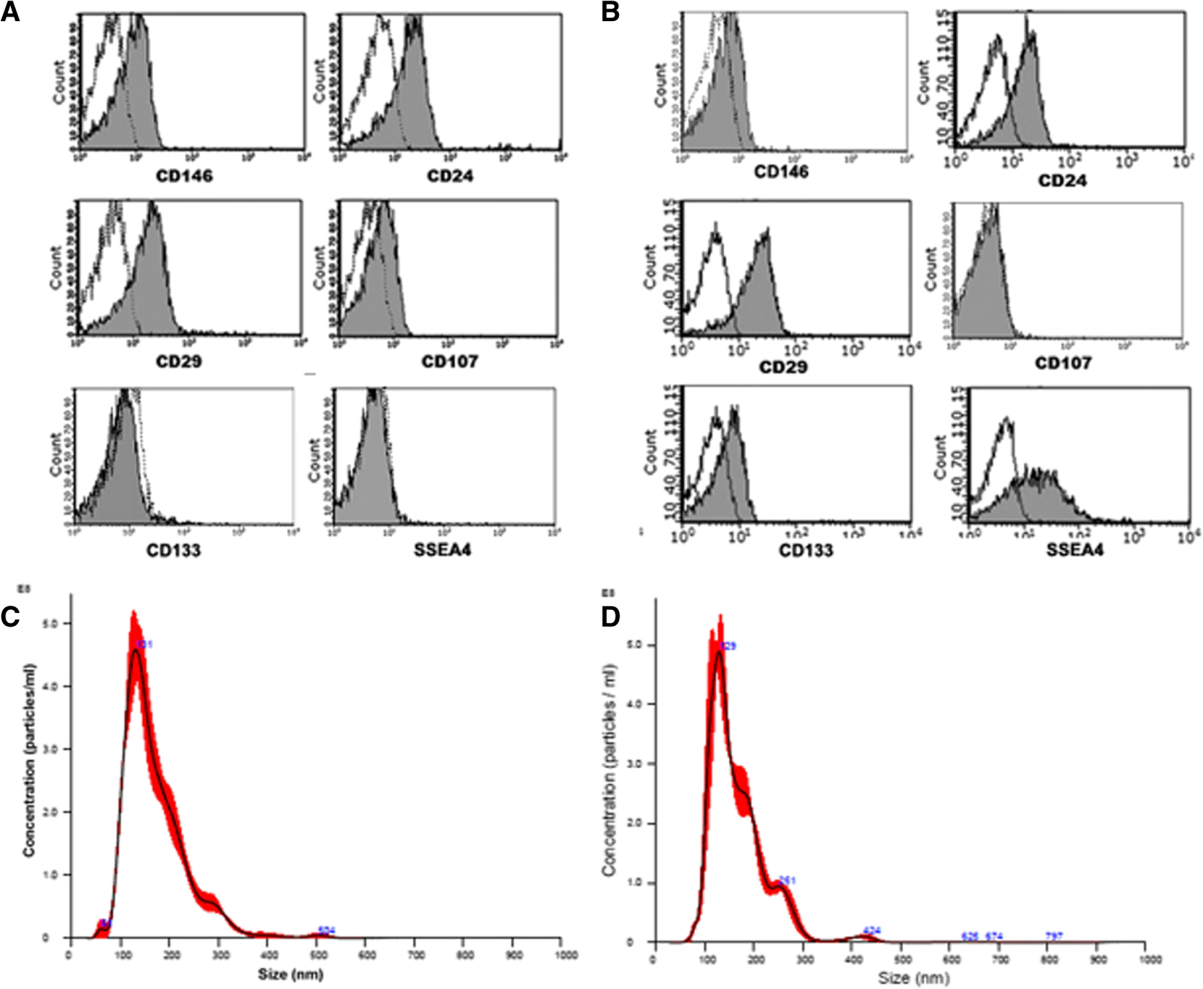
Characterization of EV surface markers and size. Representative FACS analyses of the expression of specific mesenchymal stromal cell and renal progenitor cell markers by Gl-MSC-EVs (**a**) and T-CD133+-EVs (**b**). White-filled histograms indicate the isotypic controls. **c**-**d** NTA analysis of Gl-MSC-EVs (**c**) and of T-CD133^+^-EVs (**d**). Four different preparations were tested with similar results

Cytofluorimetric analyses of Gl-MSC-EVs showed the presence of several antigens expressed by Gl-MSCs (CD146, CD29 and CD24), as well as the exosomal marker CD107 (Fig. 4a). FACS analyses of T-CD133^+^-EVs revealed the expression of typical CD133^+^ cells markers including CD24 and CD133. In addition, T-CD133^+^-EVs were also positive for SSEA4, CD29 and CD146 (Fig. 4b). Nanosight analysis confirmed the average size of EVs derived from Gl-MSCs to be 170 nm with a mode of 131 nm and a standard deviation of 62 nm (Fig. 4c). Furthermore, EVs from T-CD133^+^ had an average size of 167 nm, a mode of 129 nm and a standard deviation of 63 nm (Fig. 4d).

### EVs derived from Gl-MSCs protect against IRI-induced AKI

To evaluate whether EVs derived from Gl-MSCs and T-CD133^+^ cells were protective against AKI induced by IRI, EVs were injected in mice through the tail vein immediately after surgery. Blood chemistry analysis showed that the serum creatinine and BUN levels were significantly reduced in IRI animals treated with Gl-MSC-EVs compared to ones treated with T-CD133^+^-EVs or with the vehicle alone (Fig. 1). No significant differences were observed between treatment with Gl-MSC-EVs and Gl-MSCs (Figs. 1 and 2). The treatment of IRI mice with EVs derived from human fibroblasts had no protective effect whatsoever on AKI suggesting the specific therapeutic role of EVs derived from Gl-MSCs (Figs. 1 and 2). Futhermore, on comparing IRI mice treated with Gl-MSC-EVs to IRI mice treated with vehicle alone showed that there was a marked increase in tubular proliferation in the former compared to the latter as quantified by PCNA (Fig. 3) and BrdU staining (Additional file 1: Figure S1).

**Fig. 5:**
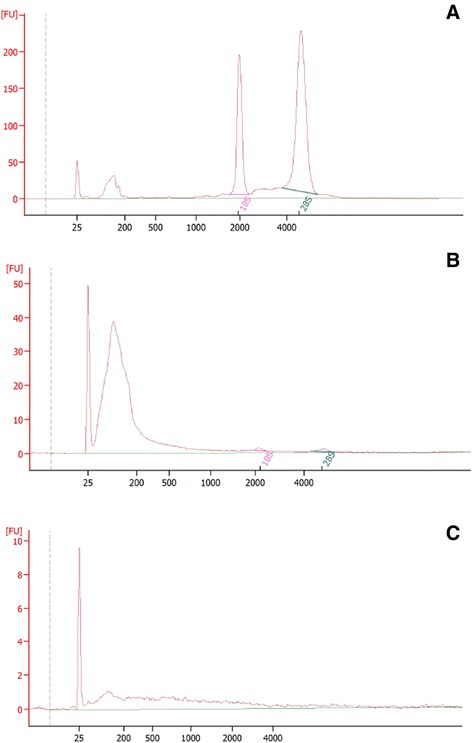
Characterization of EV RNA content. Representative bioanalyzer profiles, showing the size distribution of total RNA extracted from Gl-MSCs and Gl-MSC-EVs. The first peak (left side of each panel) represents an internal standard. The two peaks of ribosomal RNA 18S and 28S are detectable in cells (**a**) and barely detectable in the corresponding EVs (**b**). EVs exhibited a relevant peak of small RNAs. After treatment with RNase A (**c**), RNAs inside EVs were degraded

Moreover, we observed an improvement in renal function, morphology and tubular proliferation using Gl-MSC-EVs obtained by floating to exclude contamination of non-vesicular RNA and proteins comparable to that obtained with Gl-MSC-EVs (Figs. 1, 2 and 3 and Additional file 1: Figure S1). Whereas RNA in floating EVs was not reduced by physiological doses of RNase (see “Methods”), 5 μg/ml RNase was found to degrade vesicle-associated RNA as seen by bioanalyzer profiling (Fig. 5). Furthermore, RNase-treated EVs were ineffective in improving both kidney function and histological IRI recovery (Figs. 1, 2 and 3). Altogether, these results suggest that vesicular RNA was responsible for the beneficial effect of EVs in IRI mice.

### Bio-distribution of EVs in IRI-AKI mice

**Fig. 6:**
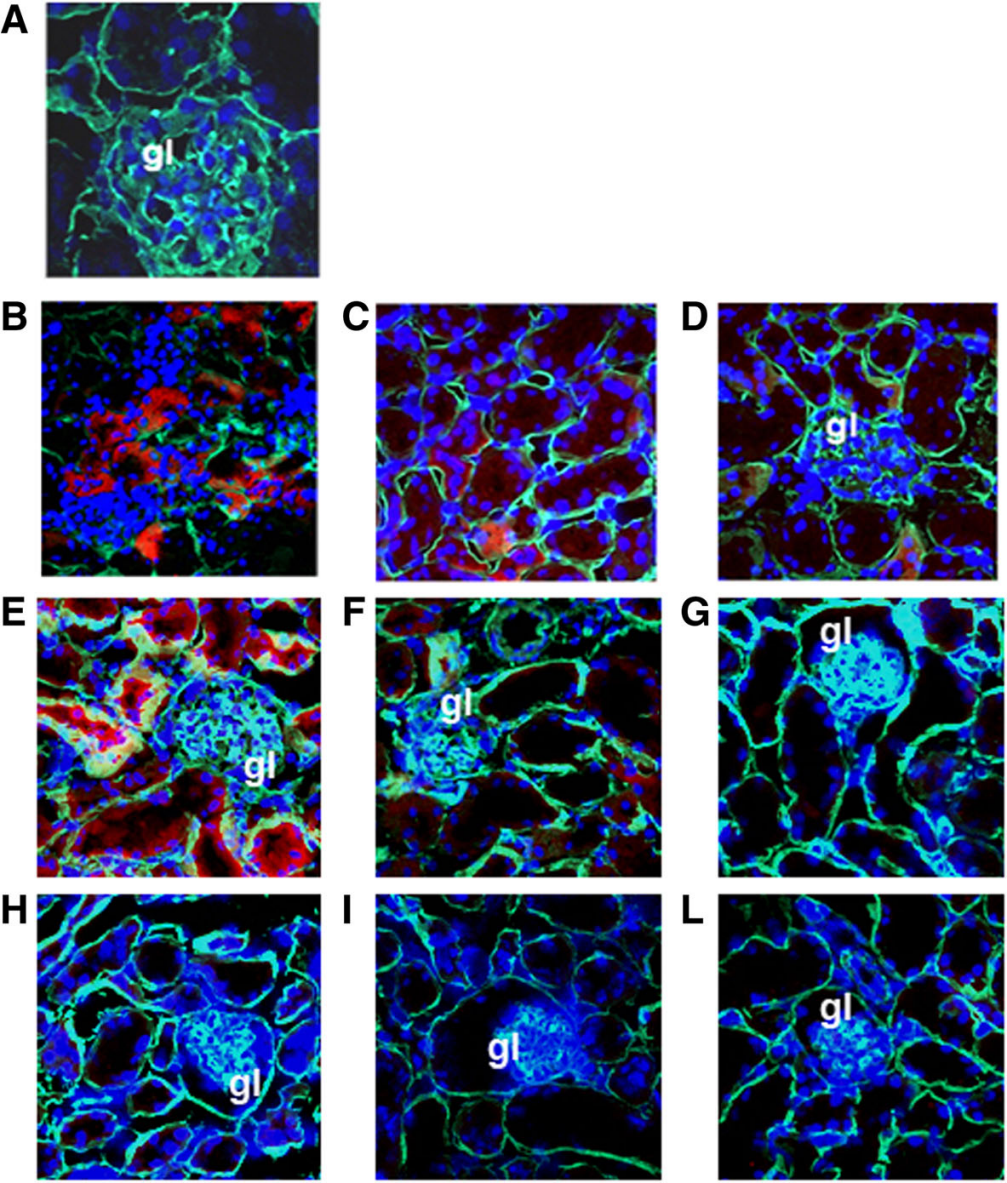
Distribution of EVs in the kidney after in vivo injection. Representative micrographs of kidney frozen tissue sections of mice injected with PKH26-labelled EVs (*red*) and stained with laminin antibody (*green*). Nuclei were stained in *blue* with Hoechst. **a** Distribution of Gl-MSC-EVs, T-CD133^+^-EVs and fibroblast-EVs in sham-operated mice. The distribution of Gl-MSC-EVs in tubules of IRI mice at 1 (**b**), 6 (**c**) and 24 (**d**) hours after administration. The distribution of T-CD133^+^-EVs in tubules of IRI mice at 1 (**e**), 6 (**f**) and 24 (**g**) hours after administration. The distribution of fibroblast-EVs in tubules of IRI mice at 1 (**h**), 6 (**i**) and 24 (**l**) hours after administration. Original magnification: ×40, except (**a**) × 63

**Fig. 7:**
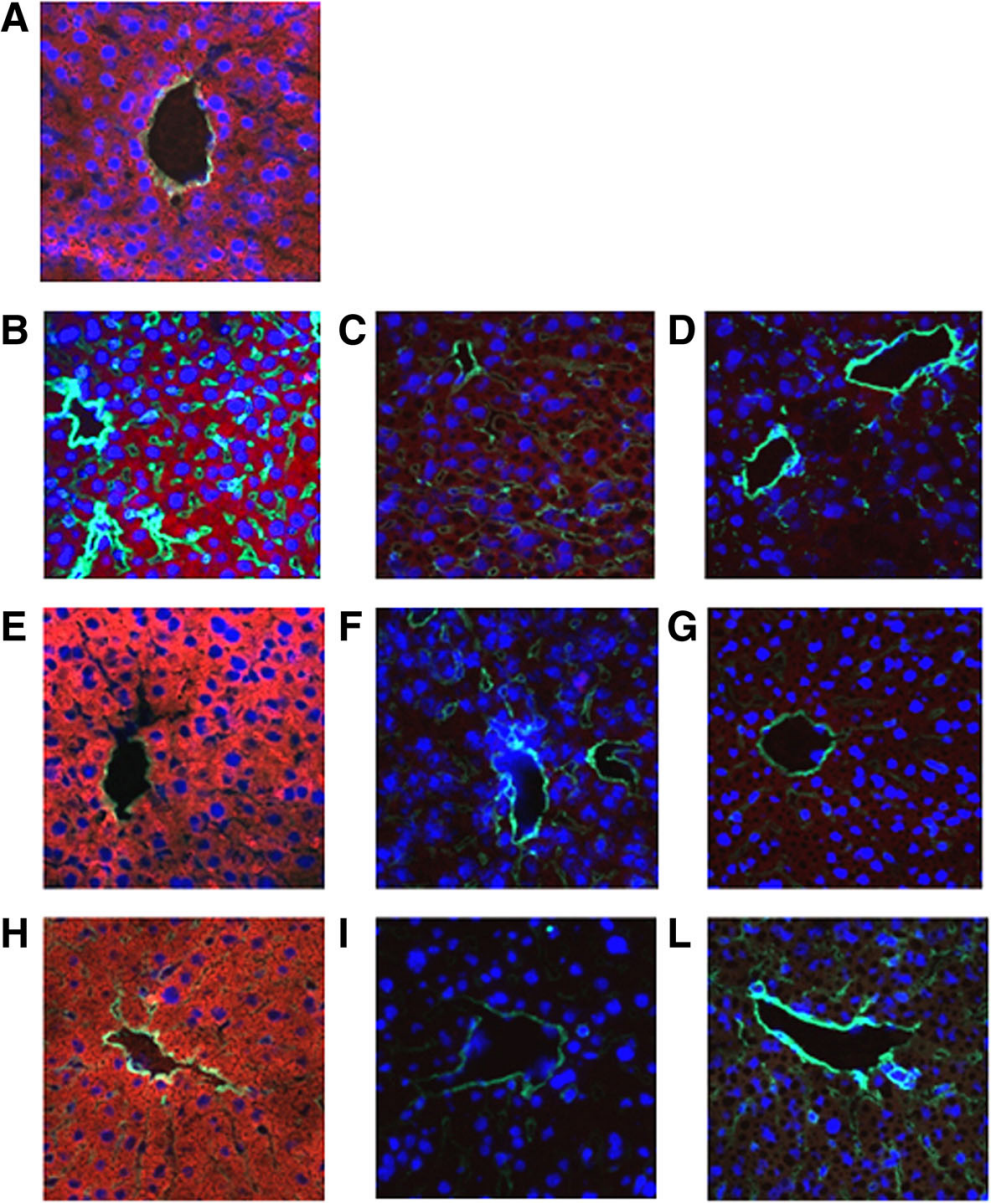
The distribution of EVs in the liver after in vivo injection. Representative micrographs of liver frozen tissue sections of mice injected with PKH26-labelled EVs (*red*) and stained with laminin antibody (*green*). Nuclei were stained in *blue* with Hoechst. **a** The distribution of Gl-MSC-EVs, T-CD133^+^-EVs and fibroblast-EVs in sham-operated mice. The distribution of Gl-MSC-EVs in the liver of IRI mice at 1 (**b**), 6 (**c**) and 24 (**d**) hours after administration. The distribution of T-CD133^+^-EVs in the liver of IRI mice at 1 (**e**), 6 (**f**) and 24 (**g**) hours after administration. The distribution of fibroblast-EVs in the liver of IRI mice at 1 (**h**), 6 (**i**) and 24 (**l**) hours after administration. Original magnification: ×40

Subsequently, the bio-distribution of EVs was checked by confocal microscopy at different time points (1, 6 and 24 hours) after injecting PKH26-labelled EVs in IRI mice. Interestingly, the labelled Gl-MSC-EVs could be detected in the kidneys of IRI mice as early as 1 hour after injection and subsequently 6 hours later especially in the tubules (Fig. 6b, c). However, 24 hours after administration only few EVs could be observed in the tubules of IRI mice (Fig. 6d). No significant accumulation of EVs was detected in the kidney of sham-operated mice (Fig. 6a). The bio-distribution observed in mice treated with Gl-MSC-EVs was also seen in mice treated with T-CD133^+^-EVs with a maximum detection of EVs in the tubules 1 hour after injection; however, this decreased at 6 hours and 24 hours subsequently (Fig. 6e-g). Although a slight amount of fibroblast-EVs were detected in the tubules 1 hour after injection, they failed to accumulate compared to the Gl-MSC-EVs and T-CD133^+^-EVs (Fig. 6 h-l). A significant buildup of Gl-MSC-EVs, T-CD133^+^-EVs and fibroblast-EVs was observed in the liver of IRI mice both at 1 and 6 hours, respectively (Fig. 7b-c, e-f, h-i) and the same in sham-operated mice as well (Fig. 7a). No EVs were detected in the liver after 24 hours post administration (Fig. 6d, g, l).

### MicroRNA profiling in Gl-MSC-EVs and comparative pathway analyses

**Fig. 8:**
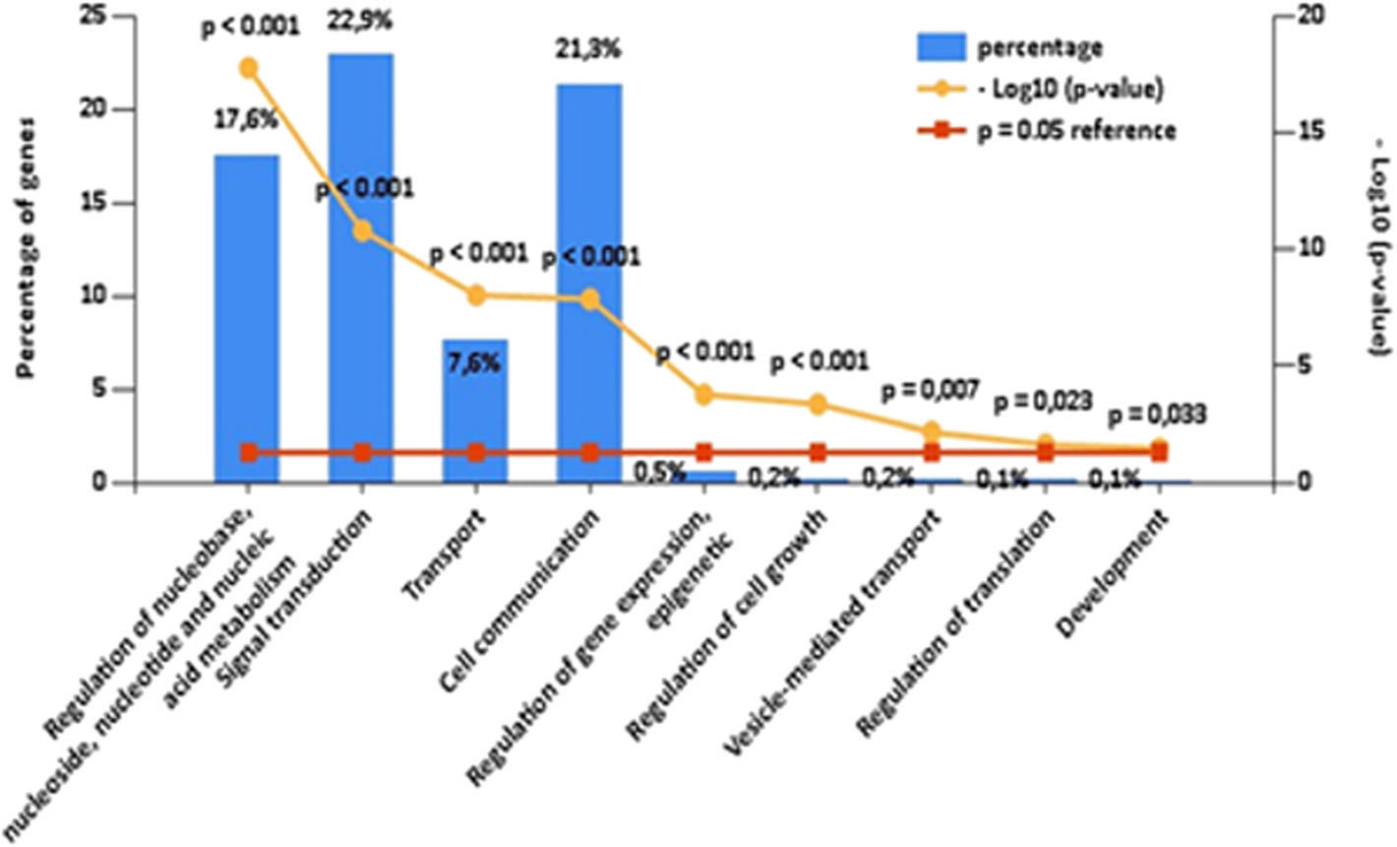
The biological process analysis of predicted target genes of miRNAs enriched in Gl-MSC-EVs. The *bars* represent the percentage of genes involved in that biological process; *red line* is the reference *p* value; *yellow line* is the relative *p* value for each category and is considered significant when *p* < 0.05

By performing a TaqMan™ array miRNA analysis we identified 62 miRNAs (Table 3) that were specifically expressed in Gl-MSC-EVs (Ct <35) and not in fibroblast-EVs (Ct >35). A target prediction analysis was performed using the Funrich tool software which identified 7318 gene targets by the selected miRNAs. The biological processes over-represented by the predicted targeted genes of miRNAs from Gl-MSC-EVs were related to nucleic acid metabolism, transport, cell communication, regulation of cell growth and gene expression (*p* <0.05) (Fig. 8).

**Table 3:** miRNAs specifically expressed by Gl-MSC EVs and not by fibroblast EVs

miRNA name	RQ
hsa-miR-299-5p	744029,35
hsa-miR-23a-3p	26432,04
hsa-miR-302b-3p	25250,07
hsa-miR-485-5p	23058,32
hsa-let-7f-5p	15771,15
hsa-miR-299-3p	11417,82
hsa-miR-654-5p	9548,11
hsa-miR-296-3p	8522,14
hsa-miR-302a-3p	5776,58
hsa-miR-139-3p	4959,57
hsa-miR-200b-3p	3600,55
hsa-miR-326	3468,27
hsa-miR-887-3p	3189,25
hsa-miR-505-3p	3017,21
hsa-miR-429	2670,70
hsa-miR-148b-3p	2176,81
hsa-miR-122-5p	2140,90
hsa-miR-449b-5p	2069,40
hsa-miR-15a-5p	1258,95
hsa-miR-215-5p	1257,20
hsa-miR-135b-5p	1162,49
hsa-miR-487a-3p	1069,71
hsa-miR-338-3p	1066,75
hsa-miR-409-5p	1033,27
hsa-miR-367-3p,	987,06
hsa-miR-23b-3p	870,08
hsa-miR-490-3p	674,18
hsa-miR-551b-3p	634,73
hsa-miR-654-3p	593,05
hsa-miR-147a	573,64
hsa-miR-548d-5p	480,04
hsa-miR-422a	467,23
hsa-miR-597-5p	410,72
hsa-miR-329-3p	397,00
hsa-miR-330-5p	353,61
hsa-miR-629-5p	313,00
hsa-miR-589-5p	247,28
hsa-miR-98-5p	211,86
hsa-miR-375	209,38
hsa-miR-545-3p	180,77
hsa-miR-146b-3p	159,56
hsa-miR-542-3p	139,68
hsa-miR-523-3p	133,81
hsa-miR-518e-3p	132,42
hsa-miR-501-3p	130,96
hsa-miR-576-5p	129,34
hsa-miR-141-3p	127,38
hsa-miR-32-5p	126,59
hsa-miR-182-5p	115,60
hsa-miR-492	102,89
hsa-miR-511-5p	102,61
hsa-miR-627-5p	98,91
hsa-miR-450b-5p	68,40
hsa-miR-517a-3p	49,45
hsa-miR-570-3p	44,82
hsa-miR-342-5p	39,97
hsa-miR-517c-3p	38,64
hsa-miR-522-3p	33,08
hsa-miR-330-3p	22,93
hsa-miR-576-3p	19,88
hsa-miR-449a	13,55
hsa-miR-369-5p	11,64

Cutoff setting: fibroblast EVs Ct >35; Gl-MSC EVs Ct <35

*Gl-MSC* glomerular mesenchymal stromal cells, *EVs* extracellular vesicles

## Discussion

In this study, we found that Gl-MSCs injected intravenously soon after kidney revascularization contributed towards reducing ischemic damage in an experimental model of IRI-induced AKI. This effect was mimicked by EVs released from Gl-MSCs suggesting their involvement in a paracrine fashion towards the beneficial effects on renal ischemic damage induced by IRI. In contrast, both CD133^+^ renal progenitor cells isolated from the tubules and their subsequent EVs did not exhibit the same protective potential on IRI-induced AKI.

The presence of MSCs in the glomeruli has been shown in both rodents and humans [43, 44]. Gl-MSCs isolated from adult human-decapsulated glomeruli were positive for both mesenchymal stem cell markers (CD146, CD105, CD44, CD73, CD29) and renal stem cell markers such as: CD24 and PAX-2. Gl-MSCs were shown to be multipotent cells with the ability to differentiate into several mesenchymal cell types such as adipocytes, osteocytes, chondrocytes, as well as specific renal cells such as podocytes and mesangial cells [44]. Moreover, Gl-MSCs derived from cross-sex transplantation demonstrated the presence of genetic phenotype of the donor kidney, suggesting that the MSCs derived were from the resident population in the kidney rather than from the bone marrow of the recipient patient [44]. Consequently, MSCs isolated from renal murine tissues also express a specific pattern of renal genes not observed in MSCs of marrow origin, therefore possibly suggesting renal MSCs to have a memory of tissue origin [50].

Several studies have demonstrated the beneficial effects of renal resident and exogenous stem/progenitor cells in repairing kidney damage after toxic or ischemic AKI [9–12, 17, 23], however, no studies have been reported on the effects of renal resident MSCs. Interestingly, the process by which both exogenous and renal resident stem/progenitor cells exert their therapeutic effects have been attributed to paracrine mechanisms [24, 26, 30, 38, 42]. Numerous reports have indicated that there was a minimal incorporation of exogenous bone marrow-derived MSCs in regenerating tubules after administration in AKI animal models [25]. Moreover, treatment with conditioned medium from MSCs was as effective as the cells. Several growth factors (VEGF [30] and IGF-1 [51]), cytokines and chemokines released by MSCs in the conditioned medium have been suggested to contribute towards this renoprotective effect [24, 29]. EVs were also found to mediate the biological effects of MSCs in several experimental models [52–57], mainly by entering the target cells through specific receptors or by membrane fusion and transferring their biologically active contents such as proteins, mRNA and miRNA. This leads to the modification of the phenotype of recipient cells by either inducing activation of molecular pathways or epigenetic reprogramming [33, 36, 41, 46, 58, 59]. Recently, several authors including our group have demonstrated the protective role of EVs derived from different sources of MSCs not only in AKI [38, 39, 60] but also in preventing the progression of AKI to CKD [61].

In the present model of IRI-induced AKI we report that T-CD133^+^ cells and T-CD133^+^-EVs were significantly less effective than Gl-MSCs and their subsequently derived EVs (GL-MSCs-EVs). Moreover, the specificity of Gl-MSCs-EVs was supported by the fact that EVs derived from fibroblasts were totally ineffective in the same setting. We also demonstrate that a single administration of Gl-MSCs during the reperfusion phase after renal ischemia significantly reduces kidney damage, stimulates renal tubular cell proliferation and leads to an improvement in overall kidney function. In addition, a similar beneficial effect was also observed when injecting a single dose of Gl-MSCs-EVs. However, on inactivating the RNA in the EVs reduced their protective effect suggesting that the biological activity observed was partly associated with the RNA content carried by EVs.

To further investigate the relevance of specific RNA shuttled by Gl-MSC-EVs, we characterized the miRNA content of Gl-MSC-EVs comparing it with fibroblast EVs that were ineffective in this AKI model. What we found was that miRNAs specifically expressed and enriched in Gl-MSC-EVs have predicted target genes involved in various biological processes such as nucleic acid metabolism, transport, cell communication, regulation of cell growth and gene expression, which could potentially influence the pro-regenerative process triggered by Gl-MSC-EVs. Although the miRNA content of EVs from different stem cell populations play an important role in renal regeneration [62], we cannot exclude the possibility of other molecules shuttled by EVs (e.g. growth factors, cytokines and chemokines) or released by cells that may also be important contributors towards the pro-regenerative effects of Gl-MSC in IRI.

Cell therapy with stem progenitor cells including exogenous MSCs has been proven to be effective in kidney repair after toxic and ischemic induced-AKI, however, this mode of therapy has also been associated with severe side effects such as long-term mal-differentiation of the engrafted cells [63] and the development of neoplastic lesions [64]. In addition, Tögel et al*.* reported that administration of bone marrow derived-MSCs in an experimental model of toxic AKI, could induce granulocytosis that worsens the intra-renal damage [65]. In addition, Burger et al. also demonstrated that injecting human umbilical cord-derived progenitor CD133^+^ cells in ischemic induced-AKI unexpectedly exacerbated the kidney damage [66]. However, in our study we did not find any malignant lesions or mal-differentiation of the engrafted cells in the renal parenchyma 3 weeks after cell administration.

## Conclusions

In conclusion, in this study we demonstrate for the first time that Gl-MSCs may contribute towards kidney repair after ischemic AKI. The mechanism can at least in part be ascribed to the release of EVs that are able to mimic the effect of Gl-MSCs.

## Additional file

Additional file 1: Figure S1. Renal cell proliferation in IRI-mice treated with Gl-MSCs-derived EVs. (A) Quantification of BrdU-positive cells/high power field (HPF) was performed in renal sections of IRI mice injected with vehicle alone (*IRI-CTL*), 400 × 10^6^ EVs produced by Gl-MSCs (*IRI-Gl-MSC-EV*), 400 × 10^6^ EVs produced by Gl-MSCs and obtained by floating process (*IRI-Gl-MSC-EV-float*), 400 × 10^6^ EVs produced by Gl-MSCs and treated with RNase (*IRI-RNase-Gl-MSC-EV*), and in sham-operated SCID mice. ANOVA with Dunnett’s multiple comparison test was performed, (^*^ *p* <0.05). (B) Representative micrographs of BrdU staining preformed on section of kidneys 2 days after IRI and treatments injection. Original magnification: ×40. (DOCX 135 kb)

## Abbreviations

AKI: Acute kidney injury
BUN: Blood urea nitrogen
CKD: Chronic kidney disease
EVs: Extracellular vesicles
Gl-MSCs: Resident population of MSCs within glomeruli
IRI: Ischemia-reperfusion injury
MSCs: Mesenchymal stromal cells
PCNA: Proliferating cell nuclear antigen
T-CD133^+^: CD133^+^ progenitor cells isolated from human tubules of the renal cortical tissue
