# Supplementary material for: The effects of glomerular and tubular renal progenitors and derived extracellular vesicles on recovery from acute kidney injury
Source: Stem Cell Res Ther. 2017 Feb 7;8:24. doi: 10.1186/s13287-017-0478-5 (PMC5297206; doi:10.1186/s13287-017-0478-5)
Supplement: Additional file 1: Figure S1. — Renal cell proliferation in IRI-mice treated with Gl-MSCs-derived EVs. (A) Quantification of BrdU-positive cells/high power field (HPF) was performed in renal sections of IRI mice injected with vehicle alone (IRI-CTL), 400 × 106 EVs produced by Gl-MSCs (IRI-Gl-MSC-EV), 400 × 106 EVs produced by Gl-MSCs and obtained by floating process (IRI-Gl-MSC-EV-float), 400 × 106 EVs produced by Gl-MSCs and treated with RNase (IRI-RNase-Gl-MSC-EV), and in sham-operated SCID mice. ANOVA with Dunnett’s multiple comparison test was performed, (* p <0.05). (B) Representative micrographs of BrdU staining preformed on section of kidneys 2 days after IRI and treatments injection. Original magnification: ×40. (DOCX 135 kb) [file 13287_2017_478_MOESM1_ESM.docx]

**
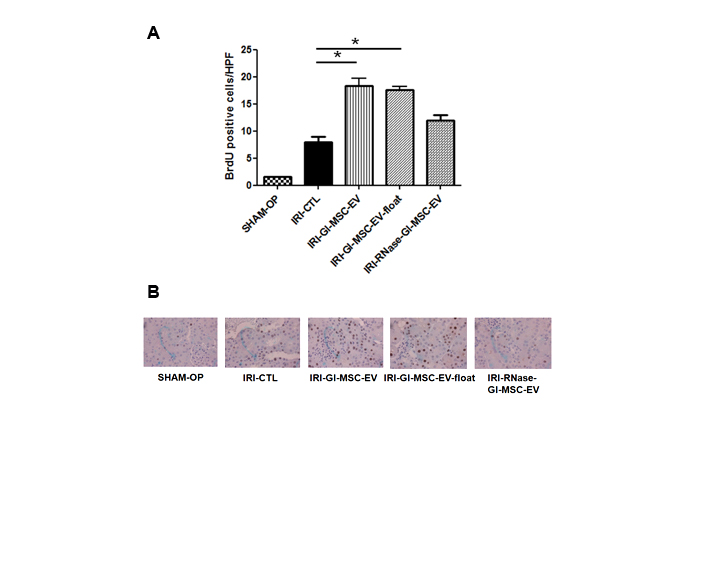
Additional file 1: Figure S1. Renal cell proliferation in IRI-mice treated with Gl-MSCs-derived EVs.** A) Quantification of BrdU-positive cells/high power field (HPF) was performed in renal sections of IRI mice injected with vehicle alone (IRI-CTL), 400x10^6^ EVs produced by Gl-MSCs (IRI-Gl-MSC-EV), 400x10^6^ EVs produced by Gl-MSCs and obtained by floating process (IRI-Gl-MSC-EV-float), 400x10^6^ EVs produced by Gl-MSCs and treated with RNase (IRI-RNase-Gl-MSC-EV), and in sham-operated SCID mice. Anova with Dunnet’s multicomparison test was performed, (*p<0.05). B) Representative micrographs of BrdU staining preformed on section of kidneys two days after IRI and treatments injection. Original magnification: 40X.
